# Enhancement of tripartite synapses as a potential therapeutic strategy for Alzheimer’s disease: a preclinical study in rTg4510 mice

**DOI:** 10.1186/s13195-019-0530-z

**Published:** 2019-08-23

**Authors:** Joshua B. Foster, Rashelle Lashley, Fangli Zhao, Xueqin Wang, Nydia Kung, Candice C. Askwith, Lin Lin, Michael W. Shultis, Kevin J. Hodgetts, Chien-Liang Glenn Lin

**Affiliations:** 1 Department of Neuroscience, College of Medicine, The Ohio State University, Columbus, OH USA; 2 Department of Neurology, Brigham and Women’s Hospital, Harvard Medical School, Cambridge, MA USA

**Keywords:** Alzheimer’s disease, Glutamate transporter EAAT2, Tauopathy, Small-molecule, rTg4510

## Abstract

**Background:**

The lack of effective treatment options for Alzheimer’s disease (AD) is of momentous societal concern. Synaptic loss is the hallmark of AD that correlates best with impaired memory and occurs early in the disease process, before the onset of clinical symptoms. We have developed a small-molecule, pyridazine-based series that enhances the structure and function of both the glial processes and the synaptic boutons that form the tripartite synapse. Previously, we have shown that these pyridazine derivatives exhibit profound efficacy in an amyloid precursor protein AD model. Here, we evaluated the efficacy of an advanced compound, LDN/OSU-0215111, in rTg4510 mice—an aggressive tauopathy model.

**Methods:**

rTg4510 mice were treated orally with vehicle or LDN/OSU-0215111 (10 mg/kg) daily from the early symptomatic stage (2 months old) to moderate (4 months old) and severe (8 months old) disease stages. At each time point, mice were subjected to a battery of behavioral tests to assess the activity levels and cognition. Also, tissue collections were performed on a subset of mice to analyze the tripartite synaptic changes, neurodegeneration, gliosis, and tau phosphorylation as assessed by immunohistochemistry and Western blotting. At 8 months of age, a subset of rTg4510 mice treated with compound was switched to vehicle treatment and analyzed behaviorally and biochemically 30 days after treatment cessation.

**Results:**

At both the moderate and severe disease stages, compound treatment normalized cognition and behavior as well as reduced synaptic loss, neurodegeneration, tau hyperphosporylation, and neuroinflammation. Importantly, after 30 days of treatment cessation, the benefits of compound treatment were sustained, indicating disease modification. We also found that compound treatment rapidly and robustly reduced tau hyperphosphorylation/deposition possibly via the inhibition of GSK3β.

**Conclusions:**

The results show that LDN/OSU-0215111 provides benefits for multiple aspects of tauopathy-dependent pathology found in Alzheimer’s disease including tripartite synapse normalization and reduction of toxic tau burden, which, in turn, likely accounted for normalized cognition and activity levels in compound-treated rTg4510 mice. This study, in combination with our previous work regarding the benefit of pyridazine derivatives against amyloid-dependent pathology, strongly supports pyridazine derivatives as a viable, clinically relevant, and disease-modifying treatment for many of the facets of Alzheimer’s disease.

**Electronic supplementary material:**

The online version of this article (10.1186/s13195-019-0530-z) contains supplementary material, which is available to authorized users.

## Background

Alzheimer’s disease (AD) is a devastating, progressive neurodegenerative disease that affects approximately 50 million people worldwide and still lacks effective therapeutic options [1, 2]. Current clinical therapies (i.e., cholinesterase inhibitors and *N*-methyl-d-aspartate (NMDA) receptor antagonists) only modestly slow the disease progression [3–5]. AD is characterized by the extracellular deposition of the amyloid β proteins in the form of plaques and the intracellular aggregation of tau proteins in the form of filaments [6–8]. Therapeutic approaches have been developed to enhance brain clearance of accumulated amyloid β and aberrant tau aggregation [9–13]; however, these approaches have not yet demonstrated success in humans. There is an imminent need to develop novel therapeutics for AD.

Glutamatergic neurons, located in the frontal, temporal (especially the hippocampus), and parietal cortices, are severely affected in AD patients [14, 15]. Studies in AD brains indicate that cognitive deficits are more highly correlated with loss of glutamatergic synapses than with neurofibrillary tangles or amyloid β burden [16–18]. Several components of the glutamate cycle, including reduced glutamate uptake activity, are disrupted in AD and are associated with cognitive decline [19–22]. Multiple lines of evidence indicate that glutamate-mediated hyperactivity of the memory network, particularly the hippocampal regions, precedes AD pathology. For example, elevated hippocampal activation was observed in individuals at risk for AD, including carriers of ApoE4, carriers of genetic mutations in familial AD, and patients with mild cognitive impairment [23–27]. In addition, seizures and epileptiform activity were found to be associated with the onset of cognitive decline and precede or coincide with the diagnosis of mild cognitive impairment or AD [19, 28]. Preventing excess glutamate-mediated toxicity and hyperexcitability is a potential therapeutic target.

Excitatory amino acid transporter 2 (EAAT2) is primarily localized in perisynaptic processes of astrocytes, closely associated with excitatory synaptic contacts [29]. EAAT2 plays a critical role in the maintenance of low extracellular glutamate levels [30, 31]. EAAT2 also plays an essential role in cognitive memory functions [32–34]. Notably, loss of EAAT2 protein and function is commonly found in AD patients and is an early event in disease pathology [19, 35, 36]. To determine whether the loss of EAAT2 contributes to AD, Mookherjee et al. crossed mice lacking one allele for EAAT2 with AβPPswe/PS1ΔE9 mice and found that the crossed mice exhibited accelerated cognitive deficits [37]. In addition, we previously crossed EAAT2 transgenic mice, which express ~ 1.5–2-folds more EAAT2, with APP_Sw,Ind_ mice [38]. The crossed mice, which had normal EAAT2 protein levels and function, exhibited significantly reduced spontaneous seizure-mediated premature death, improved cognitive functions, restored synaptic integrity, and reduced amyloid deposition, compared with their APP littermates. These studies suggest that enhanced EAAT2 function is a potential therapeutic strategy for AD.

Expression of EAAT2 protein is highly regulated at the translational level, and this translational control mechanism plays a critical role in the regulation of synaptic activity [33, 39, 40]. We previously executed high-throughput screening to search for compounds that increase EAAT2 expression through translational activation [41]. A pyridazine-based lead series was identified [42]. Studies on the mechanism of action reveal that pyridazine derivatives activate local translation of a subset of transcripts, including EAAT2, in perisynaptic astrocytic processes (PAP). This results in rapidly upregulating a subset of PAP proteins and enhancing the plasticity of the PAP [41, 43]. Importantly, enhanced plasticity of PAP subsequently leads to increased synaptic protein expression in the synapse and strengthened synaptic long-term potentiation (LTP) [41, 43]. Thus, pyridazine derivatives are capable of enhancing the structural and functional plasticity of tripartite synapses.

We previously assessed the efficacy of a compound, LDN/OSU-0212320, from the series in APP_Sw,Ind_ mice [38]. We found that this compound normalized EAAT2 expression and provided profound efficacy by preventing the development of progressive disease pathology and reversing preexisting behavioral, synaptic, and biochemical pathologies. To further develop this compound series towards a clinical application for AD, we assessed the efficacy of an advanced compound, LDN/OSU-0215111, in rTg4510 mice—a mouse model that exhibits tau-mediated AD-like pathology. rTg4510 mice harbor the familial MAPT*P301L mutation [44, 45]. Previous reports indicate that rTg4510 mice, like APP models, exhibit increased glutamate release, decreased glutamate clearance, and hyperexcitability in the hippocampus before neuron loss or tangle deposition [46]. Here, we report the findings of this efficacy study. We found that LDN/OSU-0215111 treatment in rTg4510 mice almost completely normalized pathological phenotypes at the moderate disease stage and continued to provide protection at the severe disease stage. The benefits were sustained 1 month after treatment cessation, indicating disease modification. We also found that compound treatment directly reduced toxic forms of mutant phosphorylated tau (pTau) and deposition through inhibition of the tau kinase glycogen synthase kinase-3 beta (GSK3β). The current study, in combination with our previous work, suggests that pyridazine derivatives have potential in the treatment of AD.

## Methods

### Animals

In all experiments, we utilized rTg4510 mice (Jackson; Stock #024854). MAPT*P301L expression requires the presence of two transgenes [45, 47]. The mouse prion protein promoter mediates the expression of the four-repeat microtubule-associated form of tau harboring the P301L mutation associated with familial frontal temporal dementia with Parkinsonism-17. The MAPT*P301L transgene expression is further regulated by a tetracycline-responsive element upstream of the mouse prion promoter, and therefore, its expression can be controlled in a temporal and cell type-specific manner. Transgenic expression of the tetracycline-controlled transactivator protein (tTA) is necessary to allow transcription of MAPT*P301L. The expression of tTA is effectively limited to neurons in the forebrain as it is regulated by the CamKII promoter. Doxycycline (DOX) can be used to substantially reduce mutant tau expression. However, we chose not to treat mice with DOX. Control mice were either non-transgenic or only expressed the tTA transgene, to avoid any potential leakiness of the MAPT*P301L transgene. Mice were housed in a 12-h light/dark cycle with access to food and water ad libitum except in two circumstances where mice were partially food deprived. During deprivation, mice were given sufficient food to maintain 85% of their body weight. Food deprivation occurred (1) during a 2-day period prior to the start of compound treatment to train the mice to consume honey emulsion and (2) during the T-maze task. Mice were sacrificed, and the forebrain, hippocampus, and prefrontal cortex of mice were collected and processed as described. All experiments were approved by the Institutional Animal Care and Use Committee of The Ohio State University and the National Institutes of Health Guide for the Care and Use of Laboratory Animals.

### Study design

Initially, a cohort of rTg4510 mice were crossed to EAAT2 overexpression mice [48] to determine if EAAT2 overexpression alone modulated disease state. At 4 months of age, rTg4510 × EAAT2 mice were submitted to behavioral testing and the brains were collected for pathology analysis. For the compound study, rTg4510 mice were treated daily with vehicle or LDN/OSU-0215111 (10 mg/kg) from the early symptomatic stage (2 months old) to moderate (4 months old) and severe (8 months old) disease stages. At 8 months of age, a subset of rTg4510 mice treated with the compound was switched to vehicle treatment and analyzed behaviorally and biochemically 30 days later. At each time point for each cohort, all mice were subjected to a battery of behavioral tests to assess the activity levels and cognition. Concurrently at each time point, a subset of mice was sacrificed and the brains were collected and processed as described to analyze the tripartite synaptic changes, neurodegeneration, gliosis, and tau phosphorylation as assessed by immunohistochemistry and Western blotting.

### Drugs/compounds

The present study was completed utilizing an advanced compound, LDN/OSU-0215111, derived from a pyridazine structure. We previously performed efficacy studies and a structure-activity relationship study on an earlier, tool pyridazine compound [42, 49]. The current compound was soluble in water unlike earlier compounds that were only soluble in DMSO. LDN/OSU-0215111 was dissolved in water, warmed to 37 °C for 5 min, and mixed with pre-warmed 1% polyethylene glycol 400 (Sigma-Aldrich) and 0.2% Tween-80 (Sigma-Aldrich). Vehicle was prepared in the same manner without compound. Vehicle and compound mixtures were then emulsified in warm 50% honey and 10% hydroxypropyl-β-cyclodextrin (Sigma-Aldrich) in ddH_2_O. Compound or vehicle was administered daily at 10 mg/kg as a single oral dose via pipette based on individual body weight (measured once per week).

### Doxycycline treatment

To assess if mutant tau expression correlates with increased EAAT2 observed in the present study, a cohort of mice were treated with doxycycline (DOX). rTg4510 mice were produced as described above. At 2 months old, a cohort of rTg4510 mice was switched from standard chow to DOX chow (Bio-Serv doxycycline grain-based rodent diet, 200 mg/kg) similar to a previously described protocol [50]. Mice were allowed to freely access DOX chow until the end of the study 3 weeks later. A cohort of littermates (control and rTg4510) remained on normal rodent chow for comparison. Tissues were collected, processed, and analyzed as described.

### Gliosome/synaptosome isolation

To assess protein content of perisynaptic astrocytic processes and synaptic boutons of neurons, one hemisphere of the mouse forebrain was subjected to a gliosome preparation. The procedure was performed as previously described [43]. Gliosome and synaptosome fractions were collected after the last cleanup spin and re-suspended in 1× PBS with 1× protease inhibitor (Pierce). Relative protein expression of EAAT2 was analyzed using Western blotting.

### Postsynaptic density isolation

Hippocampal postsynaptic densities were isolated as previously described [51, 52]. Briefly, one hippocampus was dissected, homogenized for 30 strokes in 500 μL of homogenization buffer, and passed through a 26-gauge needle ten times. A sample of total cell lysate was collected. The remaining total lysate was spun at 1000×*g* for 10 min, and the supernatant (S1) was collected. S1 protein concentration was assessed by Bradford protein assay. An equal amount of S1 protein was loaded, and the total volume was normalized between samples with homogenization buffer. Samples were spun at 10,000×*g* for 15 min followed by the removal of supernatant and resuspension of P2 in 100 μL homogenization buffer. Ten percent of P2 was collected (crude membrane fraction) to assess the region-specific expression of EAAT2. The remaining P2 was incubated on ice in 1.4 mL of extraction buffer (containing 0.5% Triton X-100). Samples were then centrifuged in a Beckman L8-55 M ultracentrifuge (SW 60 Ti swinging-bucket rotor) at 32,000×*g* for 20 min. The S3 supernatant was removed and collected, and the P3 pellet (postsynaptic densities) was resuspended in 100 μL of TE buffer containing 1× protease and phosphotase inhibitor (Pierce). Samples were analyzed by Western blot by loading the same volume for each sample.

### Sarkosyl extraction

Sarkosyl-insoluble proteins were isolated as previously described [53]. Briefly, one hemisphere was collected and weighed. The tissue was homogenized with 10 strokes of a pestle in 10× volume of the brain weight. Samples were spun at 27,000×*g* for 20 min. The S1 supernatant was removed, and the P1 pellet was resuspended in 5× volumes (of the original brain weight) of high salt/sucrose buffer. Samples were spun at 27,000×*g* for 20 min. The S2 supernatant was collected, 150 μL of 10% Sarkosyl was added, and the volume was normalized to 1.5 mL. Samples were then incubated at 37 °C for 60 min. Samples were spun at 150,000×*g* for 60 min. The S3 supernatant was collected, and the P3 pellet was resuspended in one-half volume (of the original brain weight) of TE buffer containing 1× protease and phosphotase inhibitor (Pierce). The preparation yielded P3 fractions that were composed almost exclusively of 64-kDa tau species. Samples were analyzed by Western blot by loading the same volume for each sample.

### Hippocampal extracellular biotinylation

Hippocampal biotinylation was performed exactly as previously described [43]. Briefly, the hippocampi from deeply anesthetized mice were dissected and sliced into 400-μM-thick sections (McIlwain tissue chopper). The hippocampal sections were washed three times in 1× PBS before being incubated with Ez-link Sulfo-LC-LC-biotin (1 mg/mL, Thermo Fisher), a cell impermeable biotinylation reagent, for 30 min. To quench the reaction, biotinylation reagent was removed and the samples were washed in 1× PBS, followed by 100 mM glycine, and again with 1× PBS. Plasma membrane proteins were liberated by incubation with radioimmunoprecipitation assay buffer. Subsequently, 100 mg of protein was incubated with 25 μL of Neutravidin beads (Pierce) overnight at 4 °C. Beads were collected by centrifugation and washed three times in 1× PBS. Beads were resuspended in 40 μL of 2× SDS loading buffer and heated at 95 °C for 10 min to release the protein bound to the beads. An equal volume of each sample was analyzed by Western blot analysis.

### Lipid-raft microdomain isolation

Lipid-raft microdomain isolation was performed exactly as previously described [43]. Briefly, mice were euthanized and one hemisphere of the forebrain was homogenized by 30 strokes in a class B Dounce homogenizer in 2 mL of homogenization buffer (150 mM NaCl, 25 mM MES, and 1× protease inhibitor, pH 6.5). After five passages through a 26-gauage needle, the homogenate was centrifuged at 1000×*g* for 10 min to remove cell bodies. An equal amount of protein from the resultant supernatant was then incubated with Brij-58 (1% final volume) at 4 °C for 1 h. Sucrose (40% final concentration by volume) was then added to the sample. One milliliter of the sample was loaded into an ultracentrifuge tube (Beckman) and overlaid with 1.8 mL of 30% sucrose and 1.2 mL of 5% sucrose (both resuspended in homogenization buffer). The gradients were centrifuged overnight (16 h) at 4 °C at 175,587×*g*. A total of 10 fractions (400 μL/fraction) were isolated using a pipette starting from the top, and the pellet (fraction 11) was resuspended in 400 μL of homogenization buffer. Protein readings were analyzed for each fraction. For Western blot analysis, the same amount of protein was loaded into each lane. The lipid raft marker flotillin-1 was used to identify the fractions that represent the lipid raft microdomain.

### Western blotting

Western blotting was performed as previously described [43]. Protein concentrations were determined by modified Bradford-Lowry (DC Protein Assay, Bio-Rad). Briefly, samples were loaded onto 8% SDS-PAGE polyacrylamide gels for separation. Proteins were transferred (Trans-Blot Trubo, Bio-Rad) to nitrocellulose membranes for antibody probing. The membranes were blocked in 5% non-fat dried milk in Tris-buffered saline with Tween-20 (0.1%) (TBS-T) for 30 min. After washing in TBS-T, the membranes were incubated with primary antibodies overnight, with gentle rocking, at 4 °C. The primary antibodies used in the current study include the following: EAAT2 (custom antibody, see [48]), PSD-95 (Thermo Scientific, AB_20923961), NMDAR2B (Millipore, AB_90772), synaptophysin (Cell Signaling Technology, AB_1904154), Tau5 (Invitrogen, AB_2536235), Tau-AT8 (Thermo Fisher, AB_223647), Tau-PHF1 (gift of Dr. Peter Davies, AB_2315150), Tau-MC1 (gift of Dr. Peter Davies, AB_2314773), GSK3β (Santa Cruz Biotechnology, AB_1123754), pGSK3β-ser9 (Santa Cruz Biotechnology, AB_10920410), flotillin-1 (Cell Signaling Technology, catalog# 1863S), and GAPDH (Santa Cruz Biotechnology, AB_10847862). After washing in TBS-T, the membranes were incubated in the respective secondary antibody for 1 h. The membranes were again washed in TBS-T and incubated with WesternBright (Advansta) enhanced chemiluminescence substrate according to the manufacturer’s instructions. Digital images were acquired using the ChemiDoc Imaging System (Bio-Rad). Signal intensities were analyzed for each antibody. The intensity for proteins of interest was normalized by the loading control flotillin-1 or GAPDH intensity to account for loading variability, when appropriate. Semi-quantitative protein expression was calculated as fold change relative to the control(s) for each blot.

### Long-term potentiation

Recordings for LTP were performed exactly as previously described [43]. Briefly, the brains were dissected and held in ice-cold cutting solution (250 mM sucrose, 25 mM d-glucose, 2.5 mM KCl, 24 mM NaHCO_3_, 1.25 mM NaH_2_PO_4_, 2.0 nM CaCl_2_, 1.5 mM MgSO_4_, and 1.0 mM kynurenic acid (pH 7.3–7.4). A vibratome (Leica) was used to collect coronal hippocampal slices (400 μm). The sections were maintained in a perfusion chamber filled with artificial cerebral spinal fluid (124 mM NaCl, 3 mM KCl, 24 NaHCO_3_, 1.25 mM NaH_2_PO_4_, 2.0 mM CaCl_2_, 1.0 mM MgSO_4_, and 10 mM d-glucose 10, bubbled with 95% O_2_ and 5% CO_2_, pH 7.3–7.4), for recovery for 1 h. Local field excitatory postsynaptic potentials from the stratum radiatum of CA1 were recorded after stimulation (100 μs duration, every 20 s) of the Schaffer collaterals. Input-output curves were generated for each slice to identify the stimulation intensity that evoked 50% the maximum response (approximately 0.2–0.3 mA). Theta-burst stimulation (TBS) was used to induce LTP. Each LTP experiment began with baseline recording for 5 min, followed by TBS, and recording LTP for 30 min. The evoked peak fEPSP was measured and normalized relative to the 5-min averaged baseline. Data were analyzed using Clampex 10.6 software.

### Immunohistochemistry

Immunohistochemistry (IHC) was performed as previously described [54]. The paraformaldehyde-fixed, sucrose-cryoprotected hemispheres were cut at 30 μm, in the sagittal plane, on a sliding, freezing microtome (Leica SM2010R). The free-floating sections were blocked in 1× PBS, 0.2% Triton X-100, and 5% normal goat serum for 60 min. Next, the sections were incubated in primary antibody overnight at 4 °C. Primary antibodies used include the following: NeuN (Millipore, AB_2298772, 1:100), GFAP (Sigma-Aldrich, AB_477010, 1:1000), synaptophysin (Cell Signaling Technology, AB_1904154, 1:100), Tau-MC1 (gift of Dr. Peter Davies, AB-2314773, 1:100), and Iba1 (GeneTex, AB_1240343, 1:100). The sections were then washed three times in 1× PBS for 10 min each wash. The sections were incubated in secondary antibody solution (1× PBS, 2% normal goat serum) for 60 min at room temperature; secondary antibodies (all from Fisher Scientific) include the following: goat anti-mouse Alexa Fluor 594 (AB_2534095, 1:500), goat anti-rabbit Alexa Fluor 594 (AB_2534091, 1:500), and DAPI (AB_2629482, 1:5000). Again, the sections were washed three times in 1× PBS for 10 min each wash. The sections were transferred to a frosted microscope slide (Fisher), coverslips were overlaid, and Immun-Mount (Thermo Scientific) mounting media were allowed to dry overnight. Images were collected from an Axioskop 2 plus microscope (× 10/0.25NA Achroplan, Carl Zeiss) using AxioVision (v4.8) software. Mean signal intensity for each section was recorded (ImageJ, v1.52i) by averaging across three segments of the region of interest, followed by subtraction of background signal intensity, and finally, the signal intensities of each section averaged together and converted into fold change relative to the respective control. The following ImageJ rectangle parameters (pixels) were used to measure the mean area intensity for each antibody: NeuN (CA1: 400 × 150 and DG: 100 × 100), synaptophysin (CA3: 100 × 150), GFAP (CA1: 250 × 250), Iba1 (CA1: 250 × 250), and Tau-MC1 (CA1: 250 × 250).

### Behavioral testing

Mice were subjected to a battery of behavioral tasks. Unless otherwise noted, all mice were habituated to the lighting conditions (30 lx) of the room for at least 30 min before behavioral testing. Each apparatus was cleaned with 70% ethanol between subjects to remove any scent cues that could alter behavior. For tasks that require multiple trials in the same day, mice were allowed to rest in between trials by serially assaying each animal in the cohort before assaying the same animal for the subsequent trial. Data were recorded using a digital webcam and were collected and analyzed using SMART software (Harvard Apparatus) or by hand. Recording and analysis setting for the software were identical between subjects for each respective task. For every task, mice that exhibited behavior > 2 standard deviations away from the mean were excluded from the analysis.

### Open-field behavior

To assess the locomotor activity and to test for any anxiety-like phenotypes, we utilized the open-field behavioral task. After habituation, individual mice were isolated in a new, clean cage for 10 min before beginning the assessment. For data acquisition, mice were placed into a 60 × 42 × 37 cm plastic box and allowed to freely move. Locomotor activity was recorded for 60 min, measured based on the center of mass motion tracking, and analyzed for total distance traveled.

### Y-maze

Y-maze was performed based on a previous study [38]. Mice were habituated in the dark for 30 min and then habituated to the behavioral room lighting for 10 min before beginning the task. Mice were placed in arm “A” and allowed to freely explore for 10 min. Mice were scored on alternation percentage. An alternation was defined as an animal exploring each of the three arms consecutively without reentering one of the previous two arms explored (i.e., entering “A” followed by “C” and then “B”). A trained observer scored this task. For an animal to be counted as entering an arm, all four paws had to enter the arm. Alternation percentage was calculated as the ratio of the number of alternations to the total number of turns. Animals that exhibited extreme stereotypy (i.e., only making left- or right-hand turns; defined as > 90% alternation) were excluded from the analysis.

### T-maze

T-maze was performed based on a previous study [38]. On day 1, mice were habituated to the maze by allowing them to freely explore for 5 min with a milk reward in each arm. On day 2, mice are placed opposite to the top of the “T” arms and allowed to enter their innate preference side with no milk reward. This is repeated five times to confirm which side is the innate preference. On days 3–9, the arm for each animal’s innate preference is closed and a milk reward is placed in the non-preference arm. Mice are trained four times per day against their innate preference. Mice are allowed up to 2 min to find the milk reward. If the animal does not find the milk reward, it is gently nudged to the milk reward. If an animal is nudged to or finds the milk reward, it is then blocked in with the milk reward for 30 s before being gently removed from the maze. On day 10 (probe trial day), mice are given one training run in the same manner as during training. Then, for the probe trial, the door to the innate preference arm is opened. Mice freely choose between the trained arm and the innate preference arm. Each animal is subjected to four trials, which are then converted to a percentage based on the number of correct choices (selecting a trained arm) for analysis.

### Novel object recognition task

We performed the novel object recognition task in a similar manner as previously described [38]. Immediately before testing, individual mice were moved to a new, clean cage for 10 min. Mice were then placed in an empty box with opaque walls (the same as used for open field). The mice were habituated inside the arena for 10 min per day for two consecutive days. On day 3, the mice were introduced to two identical objects placed equidistant on the opposite ends of the arena. The mice freely moved around the arena and interacted with the objects for 10 min. On day 4, one of the familiar objects from the previous day was removed and replaced by a novel object. We had previously validated that the familiar and the novel object have equal salience when simultaneously introduced to naïve mice. Mice were considered to be interacting with either object when the snout of the animal was within a 2-cm perimeter of the object. Video-recorded data for interaction time was standardized by converting to a discrimination index value where a score of 1 indicates interaction only with the novel object, 0 indicates equal interaction with either object (chance), and − 1 indicates interaction only with the familiar object. Discrimination index = (Time_novel_ − Time_familiar_)/(Time_novel_ + Time_familiar_). Mice that did not interact for a total of at least 30 s (combined) with both objects were excluded.

### Barnes maze

For the Barnes maze, we implemented a shortened protocol that was developed for AD mouse models [55]. The Barnes maze was composed of a circular, wooden platform with 20 circular holes, separated into 4 quadrants, drilled out around the edge of the platform equidistant from the center and one another. An escape box was placed underneath one hole. Eight unique features were placed around the maze for spatial differentiation. Lighting conditions were maintained at 30 lx. Each day, mice were habituated to the testing room for 30 min before training or testing. Individual mice were transferred from their home cage to a clean cage for 5 min before training. Mice were given at least a 30-min inter-trial interval during training with all mice being trained serially before subsequent training trials for the same animal in the same day. On day 1, mice were placed under a 2-L glass beaker in the center of the maze for 15 s. The beaker was then slowly moved to nudge the animal towards the hole with the escape box. Mice were given 2 min to fully enter the escape hole before being nudged into the escape hole. Mice were left in the escape box for 30 s before being returned to their home cage. On day 2, mice were placed under a 1-L opaque plastic beaker in the center of the maze for 15 s. The beaker was removed, and mice were allowed to explore the maze for 2 min. If mice had not found the escape box, they were trapped by the 2-L glass beaker and guided to the escape hole. If the animal did not enter the escape hole within 30 s, the glass beaker was used to nudge the animal into the hole where the animal was kept for 30 s before being returned to its home cage. Training trials were repeated a total of three times. On day 3, training was performed as described for day 2 but for a total of 2 trials. No training was performed on day 4. The probe trial was conducted on day 5. The escape box was removed from the escape hole. Mice were placed under a 1-L opaque plastic beaker in the center of the maze for 15 s. The plastic beaker was removed, digital video recording was started, and mice were allowed to explore the maze for 2 min. After testing, mice were returned to their home cage. Recorded video data from the probe trial were analyzed to determine latency to find the target hole and percent of time spent in the target quadrant.

### Statistical analysis

Graphs were created, and statistical analyses were performed using GraphPad Software (v5.0). All data is represented as mean ± SEM. The sample size for each group is indicated in the figure legend. For all behavior analysis, data were analyzed by one-way ANOVA followed by Tukey post hoc test. Barnes maze quadrant time and the Y-maze timeline in Fig. 2 were analyzed differently; data were analyzed by two-way ANOVA followed by Bonferroni post hoc test. For all biochemical isolations and IHC statistical analysis, each dataset was analyzed by performing a one-sample *t* test against a hypothetical value of 100 (normalized control-vehicle value) to determine if any samples were significantly different than controls. This was followed by a post hoc Student’s *t* test between the groups that were significantly different from control-vehicle. MC1 IHC was analyzed differently. Since control does not express MC1, only Student’s *t* test was applied to rTg4510 vehicle and compound-treated data.

## Results

### Improved cognitive functions and synaptic integrity in rTg4510-crossed EAAT2 mice

**Fig. 1:**
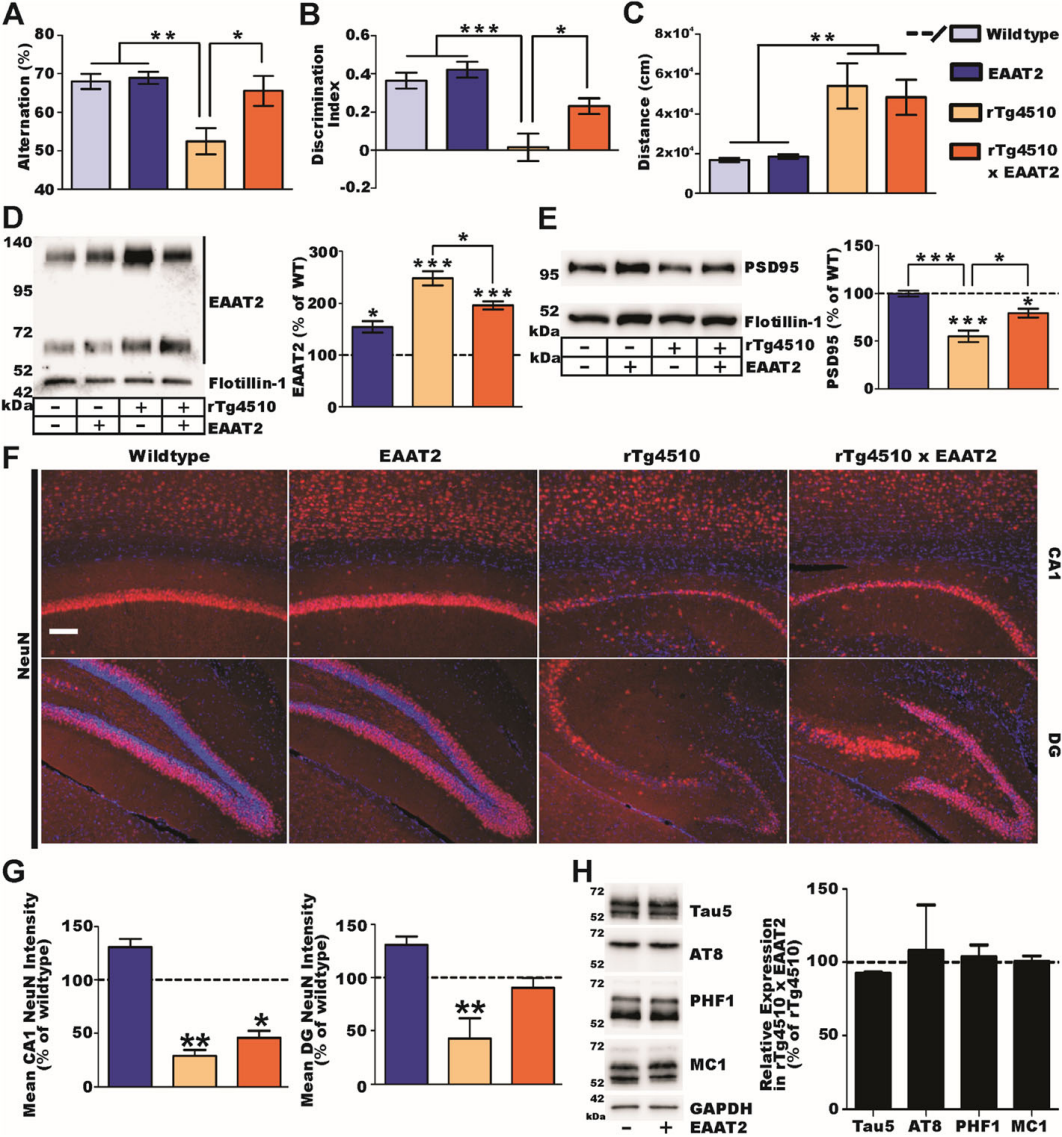
Transgenic overexpression of EAAT2 protects against mutant tau-mediated pathology. **a**–**c** Results of behavioral analysis (*n* = 13/13/10/10, respectively). rTg4510 mice show working memory deficits in the Y-maze (**a**) and recognition memory deficits in the NORT (**b**), but EAAT2 overexpression protected against these cognitive deficits. **c** However, EAAT2 overexpression did not protect against open-field hyperactivity behavior in rTg4510 mice. **d**, **e** Biochemical assessment of tripartite synapse pathology (*n* = 4/group). **d** As expected, EAAT2 transgenic mice expressed more EAAT2 in gliosomes than wild-type mice, but, unexpectedly, rTg4510 mice express even more EAAT2. rTg4510 × EAAT2 mice paradoxically exhibit reduced EAAT2 in gliosomes, partially normalizing EAAT2 towards control levels. **e** Synaptic integrity was reduced in rTg4510 mice (as assessed by PSD-95) in synaptosomes, while EAAT2 overexpression protected against synaptodegeneration. **f** NeuN immunohistochemistry showed that EAAT2 overexpression provided modest protection against neurodegeneration in rTg4510 mice. **g** Quantification of NeuN immunostaining. **h** Overexpression of EAAT2 in rTg4510 mice did not alter total tau, phosphorylated tau, or Sarkosyl-insoluble tau aggregates. *P < 0.05, **P < 0.01, *P* < 0.001

To assess the potential benefits of exclusively increased EAAT2 protein expression in rTg4510 mice, we crossed EAAT2 transgenic mice [48], which expressed 1.5–2-folds more EAAT2, with rTg4510 mice. At the age of 4 months, mice were subjected to the Y-maze test to assess the short-term memory and novel object recognition test (NORT) to assess non-spatial long-term memory. The results showed that in the Y-maze test, rTg4510 mice selected arms at random while rTg4510 × EAAT2 mice preferentially selected unexplored arms similar to the control groups (Fig. 1a). In the NORT, rTg4510 mice again selected between a novel and familiar object at random while rTg4510 × EAAT2 mice preferentially interacted with the novel object like the control groups (Fig. 1b). This demonstrated that rTg4510 × EAAT2 mice exhibited improved cognitive functions compared with their rTg4510 littermates. It was reported that rTg4510 mice exhibit hyperactivity with agitative-like behavior, which correlates with the progression of tau pathology [47]. The hyperactive phenotype has been characterized by significantly increased locomotor activity in a novel environment. Hence, we conducted open-field tests by placing the mouse in a novel box and measured the distance traveled for 1 h. The results showed that rTg4510 × EAAT2 mice still exhibited hyperactivity like their rTg4510 littermates (Fig. 1c).

Upon completion of behavioral assessment, mice were euthanized for pathological studies. To assess EAAT2 expression levels, we isolated gliosomes from the forebrain, which were enriched for perisynaptic astrocytic processes where EAAT2 is primarily localized. Western blot analysis of gliosome samples indicated that as expected, EAAT2 mice exhibited ~ 1.5-folds increase in EAAT2 protein levels, but, unexpectedly, EAAT2 protein levels were increased in rTg4510 mice ~ 3.5-folds relative to wild-type mice (Fig. 1d). Even more surprising, EAAT2 protein levels in rTg4510 × EAAT2 mice were significantly reduced relative to rTg4510 (Fig. 1d). Of note, when 2-month-old rTg4510 mice were treated with doxycycline (DOX) for 3 weeks to stop transgenic mutant tau expression, their EAAT2 protein levels were reduced in a similar manner (Additional file 1a, b), indicating that elevated EAAT2 expression was a consequence of mutant tau expression. We performed biotin labeling of cell surface proteins and found that the increased EAAT2 proteins found in rTg4510 mice were properly localized to the plasma membrane surface (Additional file 1c). In addition, we previously reported that EAAT2 is associated with the cholesterol-rich lipid raft microdomain of the plasma membrane and that the association with lipid raft is important for EAAT2 localization and function [56, 57]. We performed lipid raft preparations from the forebrains of rTg4510 mice and found that increased EAAT2 was properly localized in lipid raft microdomains as well (Additional file 1d). These results indicated that increased EAAT2 is functional and not an accumulation of inactive aggregates. For undetermined reasons, increased germline expression of EAAT2 partially normalized EAAT2 protein levels in rTg4510 × EAAT2 mice.

To assess the synaptic integrity, we isolated synaptosomes from the forebrain, which were enriched for neuronal processes. Postsynaptic density 95 (PSD-95) levels, a postsynaptic density protein, were measured by Western blot analysis. The results showed that rTg4510 mice exhibited significantly reduced PSD-95 levels in synaptosomes, suggesting a reduced number of synapses, which was normalized in rTg4510 × EAAT2 mice (Fig. 1e). Furthermore, we conducted immunohistochemical staining using NeuN antibodies to examine hippocampal neurodegeneration. The results showed a significant reduction of NeuN immunostaining in the CA1 (~ 71% reduction) and dentate gyrus (DG, ~ 57% reduction) regions of rTg4510 mice compared to their wild-type littermates (Fig. 1f, g). rTg4510 × EAAT2 littermates exhibited less neuronal loss in the CA1 (~ 54% reduction) or dentate gyrus (~ 10% reduction), suggesting improved neuronal survival (Fig. 1f, g). Of note, the efficacy observed was not due to tau transgene suppression since rTg4510 × EAAT2 mice expressed equal levels of transgenic mutant tau as their rTg4510 littermates as assessed by Western blot (Fig. 1h). In addition, we examined phosphorylated tau levels by Western blot analysis of hippocampal lysates using PHF1, AT8, and MC1 antibodies. The results showed that phosphorylated tau levels were not changed in rTg4510 × EAAT2 mice compared to their rTg4510 littermates (Fig. 1h). Taken together, these results indicate that transgenically increased EAAT2 expression in rTg4510 mice moderately protects cognitive function and synaptic integrity, while only modestly protecting against neurodegeneration, but has no effect on tau hyperphosporylation.

### LDN/OSU-0215111 principally blocked disease progression at the moderate disease stage

**Fig. 2:**
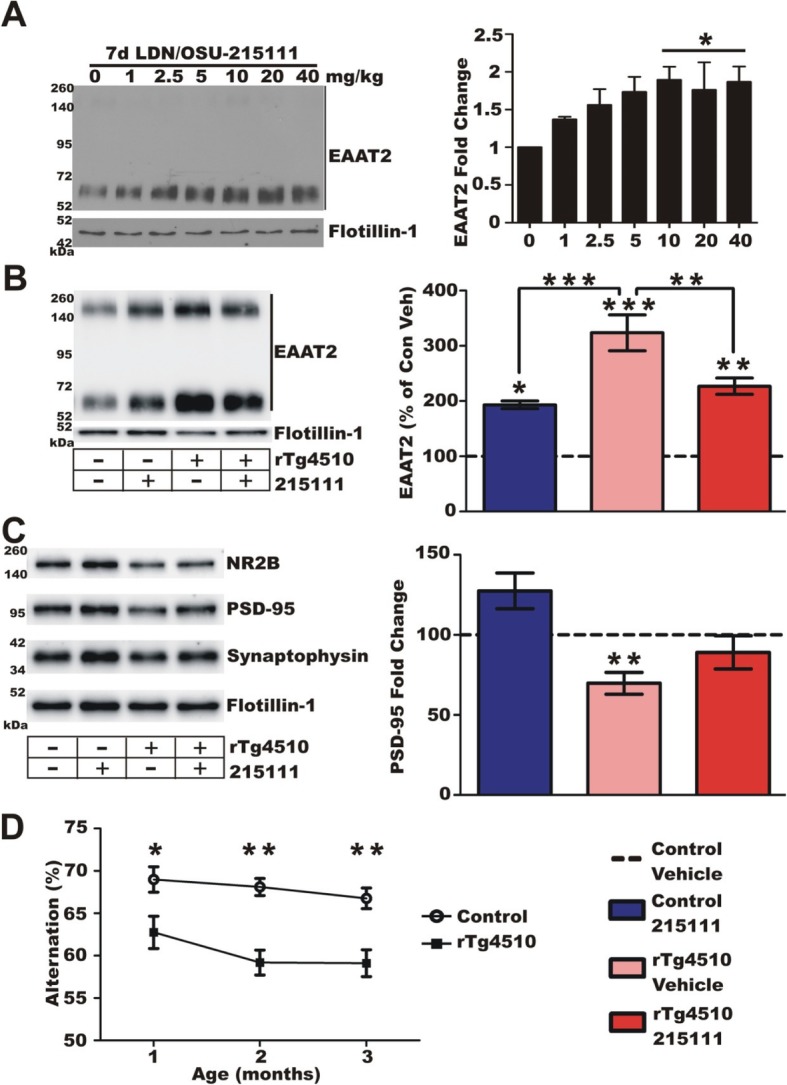
LDN/OSU-0215111 dose and treatment start time selection. **a** Control mice were treated with LDN/OSU-0215111 at doses indicated for 7 days (*n* = 3/group). Doses of 10, 20, and 40 mg/kg were found to significantly upregulate gliosome EAAT2. We therefore selected a treatment dose of 10 mg/kg for use in rTg4510 mice. **b** Similar to transgenic EAAT2 overexpression, a single compound treatment upregulated EAAT2 in gliosomes in control mice (*n* = 4/group). Likewise, compound treatment in rTg4510 mice reduced EAAT2 expression **c** rTg4510 mice exhibited a significant decrease in synaptosomal PSD-95 expression; however, a single treatment of compound normalized PSD-95 expression comparable to controls (*n* = 4/group). **d** Behavioral deficits in the Y-maze were seen at 1 month of age in rTg4510 mice which further deteriorated at 2 months of age and plateaued at 3 months (*n* = 25 and 29, respectively). We therefore chose 2 months of age for treatment start. *P < 0.05, **P < 0.01, ***P < 0.001

Next, we investigated if LDN/OSU-0215111 could provide beneficial effects in rTg4510 mice. Prior to performing the efficacy study, two preliminary experiments were conducted. First, we determined the optimal oral dose of LDN/OSU-0215111 in wild-type mice using EAAT2 as the marker. Mice were treated with the compound at 1, 2.5, 5, 10, 20, and 40 mg/kg PO daily for 7 days. Western blot analysis of gliosomes prepared from the forebrain of treated mice showed a dose-dependent increase of EAAT2 with maximal induction observed at doses of 10, 20, and 40 mg/kg (Fig. 2a). We then tested the dose of 10 mg/kg in 4-month-old rTg4510 mice. The results showed that a single dose of the compound resulted in a significant downregulation of EAAT2 (Fig. 2b), as was observed in rTg4510 × EAAT2 mice (Fig. 1d). We also assessed the effects on the synapse by examining the synaptic proteins in synaptosomes prepared from the forebrain of treated mice. The results showed that synaptic proteins, including PSD-95, NR2B (NMDA receptor subunit), and synaptophysin (pre-synaptic protein), were significantly decreased in rTg4510 mice but were partially restored following a single dose of compound (Fig. 2c). Similar results were also observed when we examined PSD-95 levels in postsynaptic density complexes prepared from the hippocampal regions. These results indicated that a single dose of LDN/OSU-0215111 at 10 mg/kg was capable of partially normalizing EAAT2 levels and restoring synaptic integrity in rTg4510 mice. We therefore chose the dose of 10 mg/kg for the studies below. Second, we determined the appropriate age to begin the treatment. We sought to identify a time point that represents the early symptomatic stage. Y-maze tests were conducted at 1, 2, and 3 months of age. We found that rTg4510 mice already exhibited impaired functioning in the test at 1 month, further deterioration at 2 months, and a leveling off between 2 and 3 months of age (Fig. 2d). We therefore determined treatment would begin at 2 months of age.

The efficacy study was conducted as follows. Littermate-matched mice with equal distribution of sexes were divided into four groups: control (wild-type)/vehicle, control/compound, rTg4510/vehicle, and rTg4510/compound (*n* = 30–35 per group). Mice received compound at 10 mg/kg/day by voluntary ingestion (PO) of compound in honey emulsion starting at 2 months of age. At 4 months old (moderate disease stage), mice were subjected to open-field tests to assess agitation-like behavior and then three cognitive tests, including Y-maze, NORT, and T-maze tests. Examiners were blinded regarding the treatment. Upon completion of behavioral assessment, a subset of mice (*n* = 10–12 per group) was euthanized for pathological studies and the rest of mice continued receiving treatment. At 8 months old (severe disease stage), mice again were subjected to open-field tests and cognitive tests. Following behavioral testing, mice were euthanized for pathological studies or electrophysiological studies.

**Fig. 3:**
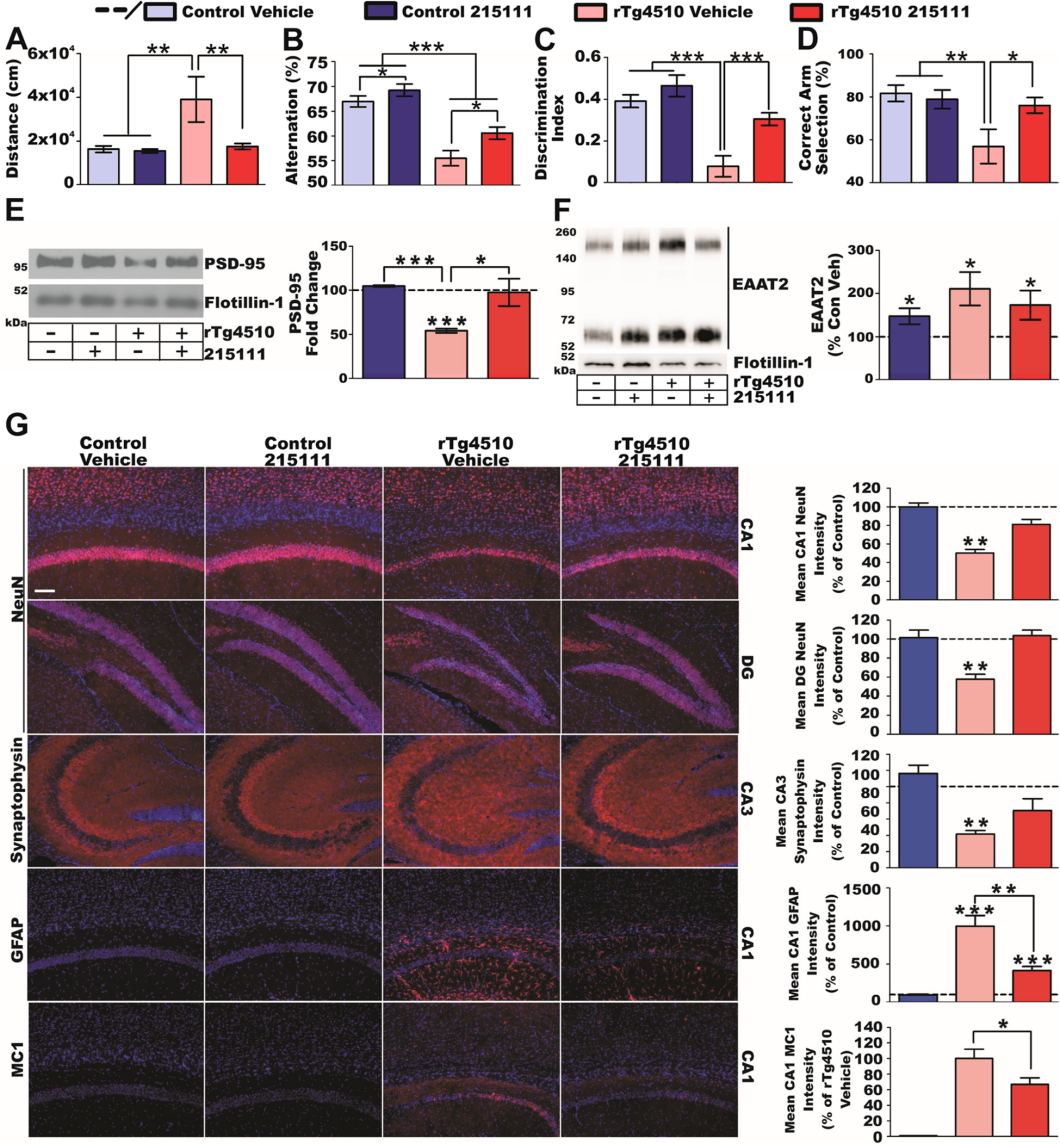
LDN/OSU-0215111 efficacy at moderate disease stage. **a**–**d** Results of behavioral battery (*n* = 27/27/23/27, respectively). Compound treatment normalized hyperactivity in the open field (**a**), short-term memory in the Y-maze (**b**), recognition memory in the NORT, (**c**) and cognition in the T-maze (**d**). **e** PSD-95 expression in hippocampal postsynaptic densities of rTg4510 was significantly reduced (*n* = 5/5/4/4, respectively), showing synaptic loss. Compound treatment in rTg4510 mice restored synaptic integrity indistinguishable from controls. **f** Hippocampal crude membrane preparations (*n* = 5/5/4/4, respectively) revealed increased EAAT2 in the rTg4510 vehicle group which was partially normalized by compound treatment. **g** Representative immunohistochemistry images of hippocampal subregions (*n* = 4 animals/group; average of ≥ 3 sections/animal). The cell nuclei were stained by DAPI (blue). Quantification (right) is percent change relative to control vehicle (dashed line), except MC1 (compared to rTg4510 vehicle). Control groups exhibited no differences. NeuN immunostaining demonstrated significant neurodegeneration in CA1 and DG of rTg4510 mice, which was prevented by compound treatment. Compound treatment maintained CA3 synaptic integrity (synaptophysin) and significantly reduced neurofibrillary tangle accumulation (MC1) in CA1 of rTg4510 mice. Finally, GFAP immunoreactivity was significantly increased in both rTg4510 groups, but compound treatment reduced gliosis. Scale bar = 100 μm. *P < 0.05, **P < 0.01, ***P < 0.001

The results of behavioral and pathological assessments at 4 months of age are presented in Fig. 3. No obvious differences were observed between males and females (male/female *n* value: control-vehicle, 15/12; control-compound, 14/13; rTg4510-vehicle, 10/13; rTg4510, 12/15). Open-field results indicated that compound treatment completely ameliorated agitative-like behavior in rTg4510 mice (Fig. 3a). In all three cognitive tests, vehicle-treated rTg4510 mice showed very significant impairments. Importantly, compound treatment significantly improved short-term memory (Y-maze; Fig. 3b), non-spatial long-term memory (NORT; Fig. 3c), and spatial learning memory (T-maze; Fig. 3d). It is notable that compound-treated rTg4510 mice were statistically indistinguishable from control mice in NORT and T-maze tasks, indicating that compound treatment almost completely prevented cognitive decline in rTg4510 mice. Consistently, follow-up pathological studies showed that PSD-95 levels in the postsynaptic density complexes prepared from the hippocampus were markedly decreased in vehicle-treated rTg4510 mice but were significantly restored to nearly normal levels in compound-treated rTg4510 mice (Fig. 3e). It is notable that synaptic PSD-95 protein loss was not found in the prefrontal cortex, suggesting that overt disease pathology is limited to the hippocampus at this stage. We examined EAAT2 levels in the hippocampus by performing crude plasma membrane preparations to assess membrane-bound EAAT2. The results showed an increase in the expression of EAAT2 in vehicle-treated rTg4510 mice as was previously observed in Figs. 1d and 2b, which were partially normalized by the compound treatment (Fig. 3f).

Furthermore, immunohistochemical analysis of hippocampal regions revealed that vehicle-treated rTg4510 mice exhibited severe neurodegeneration in the CA1 and the DG region, as assessed by NeuN immunostaining, but the neuronal loss was almost completely prevented in compound-treated rTg4510 mice (Fig. 3g). Moreover, expression of the pre-synaptic marker synaptophysin (a proxy for synaptic integrity) was completely lost in the CA3 region in vehicle-treated rTg4510 mice but was well-preserved in compound-treated rTg4510 mice (Fig. 3g). Neurofibrillary tangles, which were detected in the CA1 region of vehicle-treated rTg4510 mice by MC1 immunostaining, were significantly reduced in compound-treated rTg4510 mice (Fig. 3g). We examined astroglial activation and gliosis by GFAP (glial fibrillary acidic protein) immunostaining and found a remarkable increase in GFAP immunoreactivity in the CA1 region of vehicle-treated rTg4510 mice, which was significantly decreased in compound-treated rTg4510 mice (Fig. 3g). We also examined microglial activation by Iba1 (ionized calcium-binding adaptor molecule 1) immunostaining, but no increase in Iba1 immunoreactivity was found in rTg4510 mice at this age. Taken together, we found that when treatment began at 2 months of age, rTg4510 mice demonstrated near-normal cognition and behavior, almost indistinguishable from control mice, at 4 months old. This indicates the exceptional efficacy of LDN/OSU-0215111.

### LDN/OSU-0215111 continues to provide protection at the severe disease stage

**Fig. 4:**
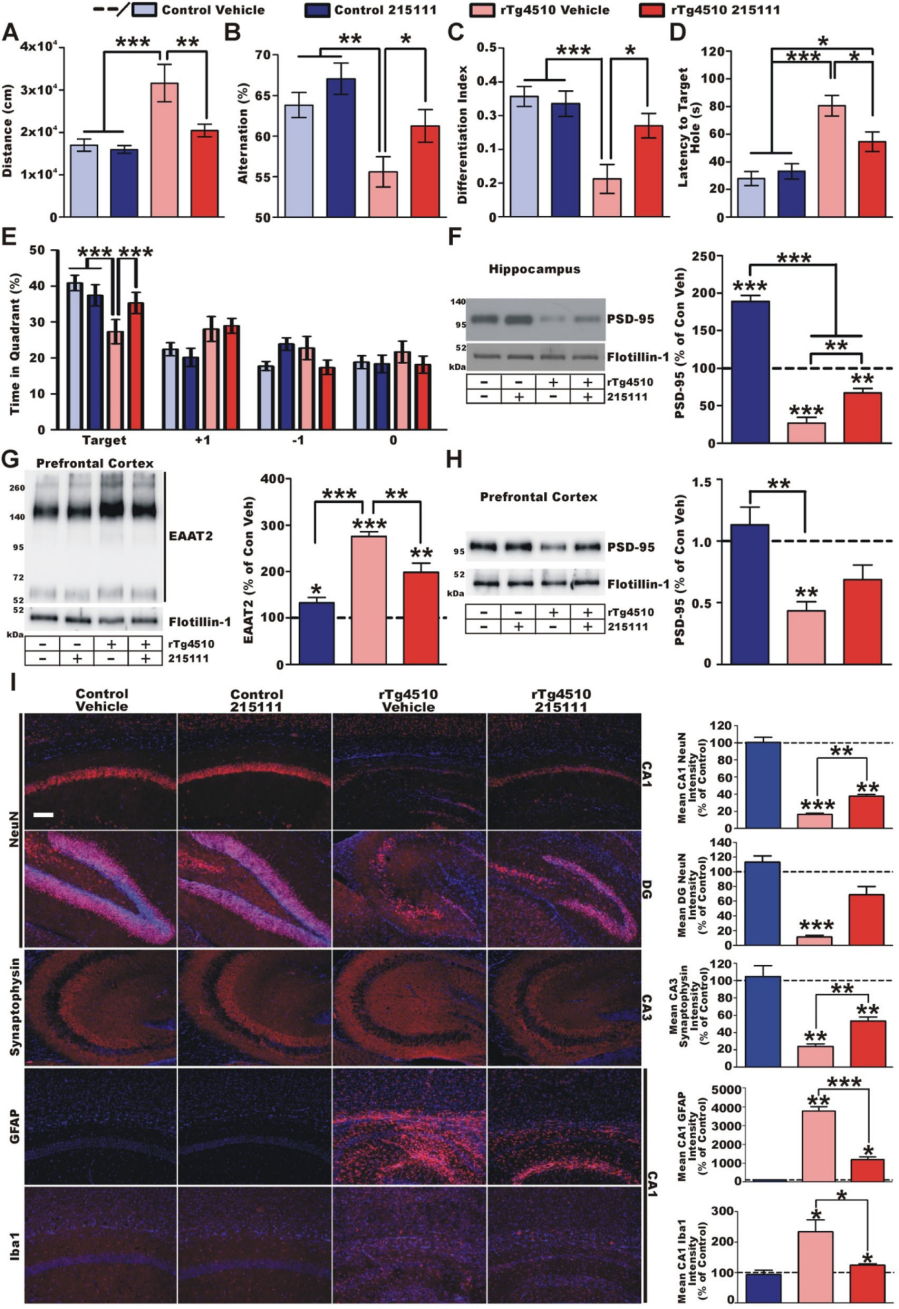
LDN/OSU-0215111 efficacy at severe disease stage. **a**–**e** Results of behavioral battery (*n* = 34/21/28/32, respectively). Long-term compound treatment continued to prevent the development of agitation-like behavior (**a**) while maintaining improved cognition in Y-maze (**b**) recognition memory in the NORT (**c**) and spatial memory in Barnes maze (**d**, **e**) in rTg4510 mice. **f** Loss of PSD-95 in rTg4510 hippocampal postsynaptic densities was robust (*n* = 8/group); however, compound treatment continued to significantly reduce synaptodegeneration. **g**, **h** PFC tripartite-synapse integrity (*n* = 4/group). Similar to the hippocampus at 4 months, rTg4510 PFC postsynaptic densities exhibit decreased PSD-95 expression (**g**) and increased crude membrane EAAT2 expression (**h**). Compound treatment partially normalized both phenotypes. **i** Representative immunohistochemistry images of the hippocampus (*n* = 4/group). Cell nuclei were stained by DAPI (blue). Quantification (right) is percent change relative to control vehicles (dashed line). Neurodegeneration (as assessed by NeuN) was observed in CA1 and DG of rTg4510 vehicle mice; however, compound treatment significantly reduced neuronal loss. A similar pattern was observed for synaptic integrity (synaptophysin). rTg4510 mice exhibit increased GFAP and Iba1 in CA1, which was reduced and partially normalized by compound treatment. Scale bar = 100 μm. *P < 0.05, **P < 0.01, ***P < 0.001

Figure 4 shows the results of behavioral and pathological assessments at 8 months old. No obvious differences were observed between males and females (male/female *n* value; control-vehicle, 19/15; control-compound, 12/9; rTg4510-vehicle, 11/17; rTg4510, 16/16). The agitative-like behavior was still normalized by compound treatment, as assessed by open-field tests (Fig. 4a). For the cognitive assessment, LDN/OSU-0215111 still significantly prevented short-term memory decline (Y-maze; Fig. 4b) and non-spatial long-term memory decline (NORT; Fig. 4c). We utilized the Barnes maze test in lieu of the T-maze test to assess spatial learning memory. Vehicle-treated rTg4510 mice took significantly longer to find the target hole (Fig. 4d) and spent significantly less time in the target quadrant of the maze (Fig. 4e). On the other hand, compound-treated rTg4510 mice found the target hole significantly faster (Fig. 4d) and spent more time in the target quadrant (Fig. 4e). These behavior studies indicated that compound treatment still provided significant beneficial effects to cognitive functions at this late stage of the disease.

Follow-up pathological studies showed that vehicle-treated rTg4510 mice exhibited very significantly reduced PSD-95 levels in the postsynaptic density complexes prepared from the hippocampi (Fig. 4f). Compound-treated rTg4510 mice maintained significantly higher PSD-95 expression than vehicle-treated rTg4510 mice. Unlike at 4 months of age, EAAT2 expression in the hippocampus showed no difference between treated and untreated rTg4510 mice. However, by 8 months, the prefrontal cortex exhibited synaptic pathology, similar to what was observed in the hippocampus at 4 months old. We found a significant increase of EAAT2 expression and a significant loss of PSD-95 protein in the prefrontal cortex of vehicle-treated rTg4510 mice (Fig. 4g, h). Compound treatment partially normalized the expression of both PSD-95 and EAAT2.

Immunohistochemical analysis of the hippocampal regions revealed that at this stage of disease progression, both vehicle- and compound-treated rTg4510 mice exhibit significant neurodegeneration in the CA1 and DG regions, as assessed by NeuN immunostaining. However, LDN/OSU-0215111-treated rTg4510 mice showed significantly reduced levels of neurodegeneration in both regions (Fig. 4i). In addition, the expression of synaptophysin was partially preserved in compound-treated rTg4510 mice (Fig. 4i). Both rTg4510 groups exhibit increased GFAP immunoreactivity; however, compound treatment significantly reduced GFAP immunoreactivity in the CA1 (Fig. 4i). Vehicle-treated rTg4510 mice had a significant increase in Iba1 immunoreactivity. The increase in Iba1 immunoreactivity was significantly lower in compound-treated mice (Fig. 4i). Overall, LDN/OSU-0215111 continues to provide disease-modifying and disease-delaying benefits against all phenotypes tested after long-term treatment.

### LDN/OSU-0215111 benefits are sustained 1 month after treatment cessation

**Fig. 5:**
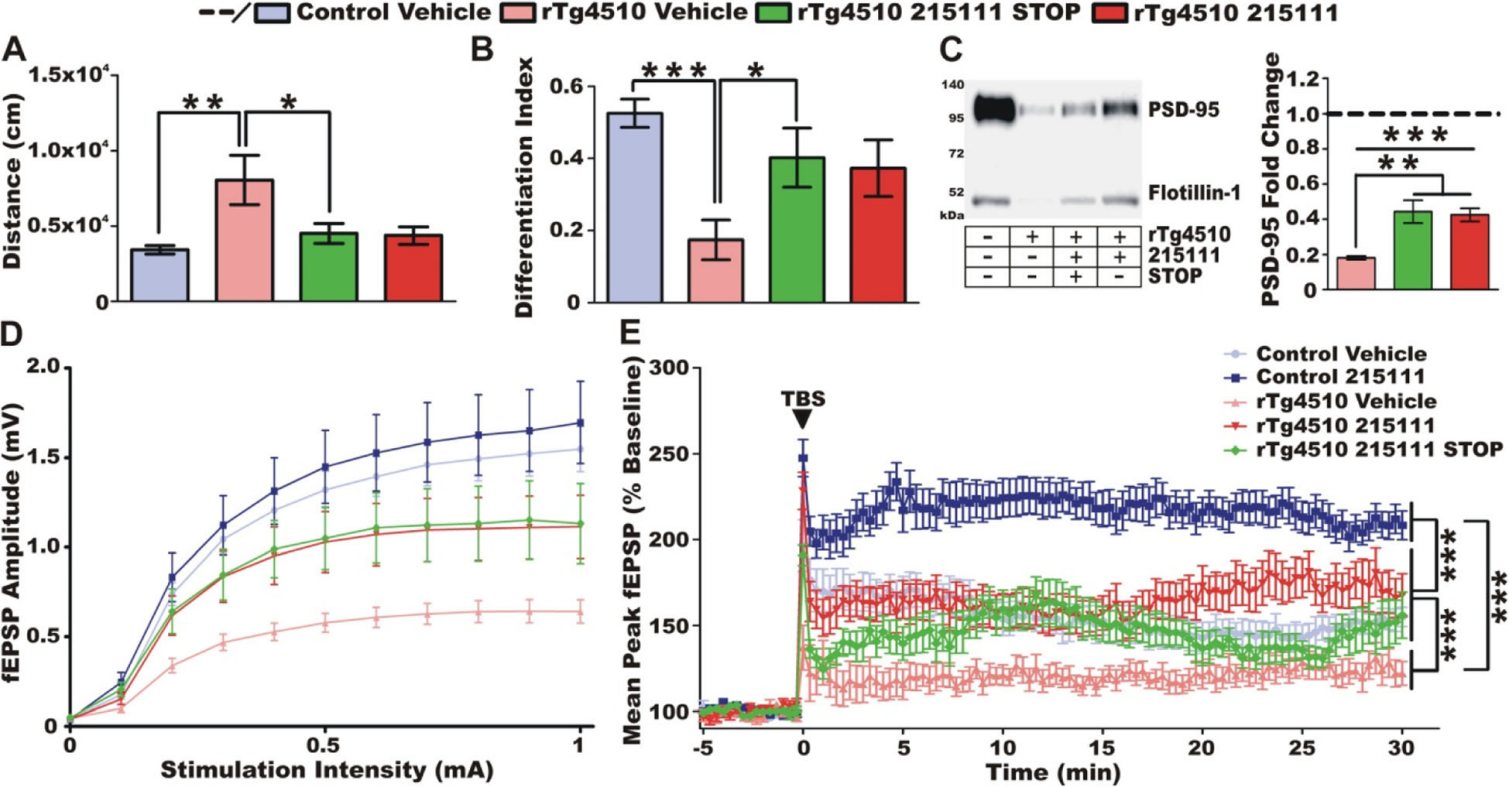
LDN/OSU-0215111 modifies disease progression. In a cohort of rTg4510 compound-treated mice, treatment was terminated, and 30 days later, behavioral analysis (*n* = 9/9/6/4, respectively), tissue collection, and LTP were performed. **a** Hyperactivity in the open field and **b** cognitive function in the NORT remained normalized in the rTg4510 treatment STOP group relative to the rTg4510 vehicle group. **c** PSD-95 protein expression in hippocampal postsynaptic densities of rTg4510 mice continued to remain significantly higher in the treatment STOP group compared to the vehicle group. **d**, **e** Hippocampal functional connectivity in the CA3-CA1 circuit at (*n* = 4/11, 4/17, 3/10, 4/14, and 2/9, respectively). **d** Input/output (IO) curves for all five groups of mice. All rTg4510 mice exhibit reduced synaptic strength compared to controls. However, both compound cessation and continuation groups show enhanced synaptic strength compared to rTg4510 vehicle mice. **e** Vehicle-treated rTg4510 mice displayed significantly reduced LTP, while the compound treatment cessation and continuation groups displayed LTP that was indistinguishable from control vehicle mice. Of note, compound-treated controls displayed significantly increased LTP relative to control vehicles. TBS, theta-burst stimulation. *P < 0.05, **P < 0.01, ***P < 0.001

To determine how long the benefits of treatment could persist, a cohort of compound-treated rTg4510 mice was switched to vehicle treatment (STOP treatment) at 8 months of age. One-month post-treatment cessation, behavior tests, followed by pathological studies, were conducted to compare the STOP treatment rTg4510 group with the continued treatment rTg4510 group, the vehicle rTg4510 group, and the control vehicle group. Surprisingly, locomotor activity levels in the open field remained normalized in the STOP treatment group (Fig. 5a). NORT results indicated that long-term memory was preserved in the STOP treatment rTg4510 mice (Fig. 5b). For both behavioral tasks, the STOP treatment and the continued treatment groups performed essentially identically. Follow-up pathological studies showed that all rTg4510 groups exhibited significant reductions in PSD-95 levels in the postsynaptic densities of the hippocampus; however, the STOP treatment group showed a significant increase, approximately twofolds, in PSD-95 levels relative to the vehicle group (Fig. 5c). The expression level of PSD-95 in the STOP treatment group was indistinguishable from the continued treatment group. These results indicated that LDN/OSU-0215111 directly modifies the disease pathology and does not act as a palliative care agent.

We further conducted electrophysiological studies to examine the integrity of the hippocampal synaptic circuit. We analyzed the changes to LTP in the hippocampal CA3-CA1 circuit along the Schaffer collateral pathway. By only looking at the I/O curves, it was clear that all three rTg4510 groups had reduced synaptic strength (Fig. 5d). However, the vehicle rTg4510 group had the most substantially reduced synaptic strength while the continued treatment and the STOP treatment groups showed an intermediate reduction. The vehicle rTg4510 group exhibited very little LTP (Fig. 5e). Both the STOP treatment and continued treatment rTg4510 groups were found to have significantly enhanced LTP relative to the vehicle rTg4510 group that was statistically indistinguishable from the control vehicle group. This is surprising because, although both continued treatment and STOP treatment groups exhibited neurodegeneration and reduced synaptic integrity relative to controls, both were able to form relatively normal LTP. Of note, compound-treated controls were found to have highly elevated levels of LTP after stimulation compared to vehicle-treated controls suggesting these mice exhibit enhanced synaptic plasticity.

### LDN/OSU-0215111 reduces tau hyperphosphorylation/deposition putatively by modifying kinase activity

**Fig. 6:**
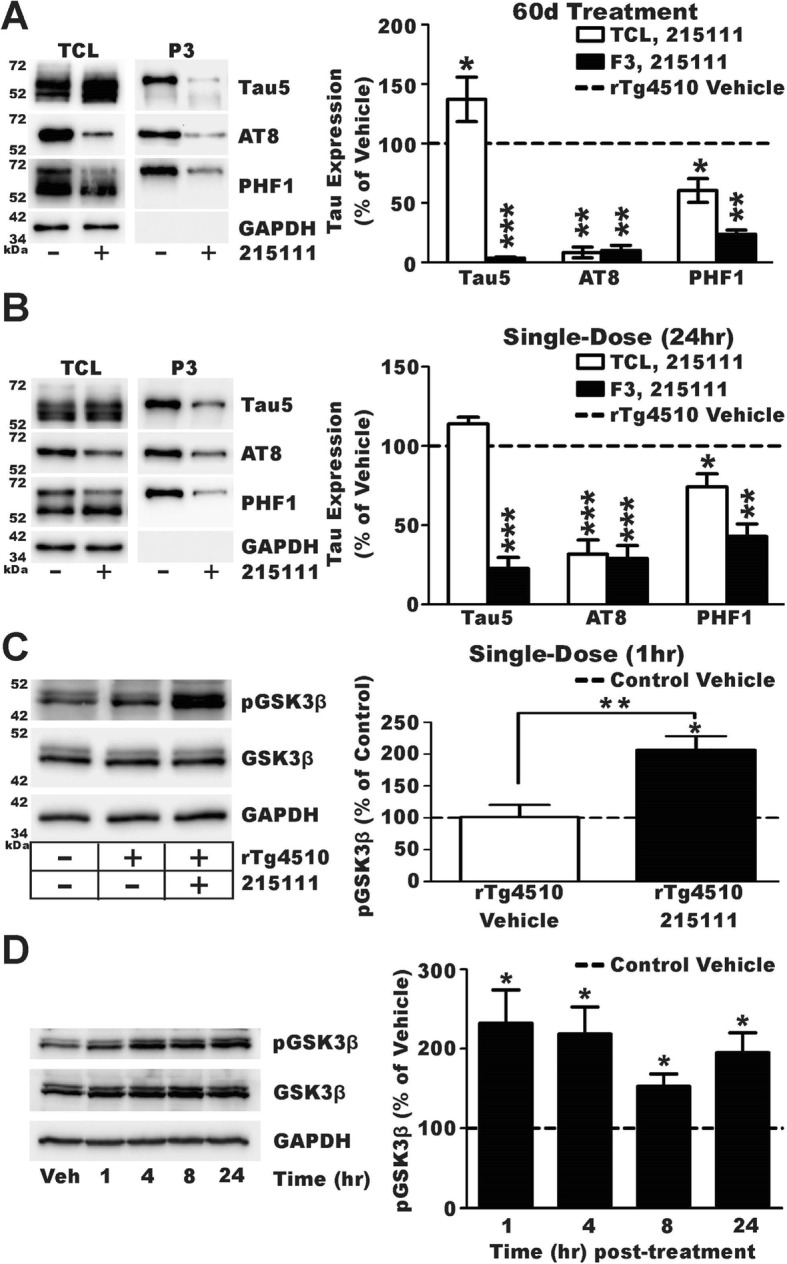
LDN/OSU-0215111 reduces pTau and inhibits GSK3β. **a**, **b** Effect of compound treatment on phosphorylated tau in total lysates (TCL, open bars) and in the Sarkosyl-insoluble (P3, black bars) fraction (*n* = 4/group). Dashed line represents the expression in the rTg4510 vehicle group. **a** Long-term compound treatment slightly, but significantly, increased total tau (Tau5) expression in the forebrain and yet reduced phosphorylated (AT8 and PHF1). There was a very significant reduction of total tau in the P3 fraction and subsequent reductions of phosphorylated tau expression. **b** Single dosing of compound did not change the total forebrain tau expression (Tau5); however, a significant reduction of phosphorylation in PHF1 and a more robust reduction of AT8 phosphorylation were observed. In the P3 fraction, total tau was reduced and, subsequently, so was all aberrant forms of tau. **c** Basal inhibitory phosphorylation of GSK3β at Ser9 in rTg4510 vehicle mice was similar to control levels (*n* = 4/group). Compound treatment significantly increased phosphorylation of GSK3β at Ser9 in rTg4510 mice approximately twofolds within 1 h of treatment, and **d** this increased GSK3β phosphorylation is maintained up to 24 h after a single dose. *P < 0.05, **P < 0.01, ***P < 0.001

As shown in Fig. 3g, we observed reduced neurofibrillary tangles in long-term compound-treated rTg4510 mice. We, therefore, investigated if the compound could reduce toxic forms of tau. We examined tau expression levels in total cell lysates (TCL) and Sarkosyl-insoluble fractions (P3) prepared from the forebrains of rTg4510 mice that were harvested at 4 months old (after 2 months of treatment) by Western blot analysis. Four antibodies, which recognized different phosphorylation sites or pathological forms of tau, were used: PHF1 recognized Ser 396 and Ser404; AT8 recognized Ser202 and Thr205; MC-1 recognized neurofibrillary tangles, and Tau5 recognized all of tau (phosphorylated and non-phosphorylated isoforms). The results showed a robust decrease in the expression of all forms of phosphorylated tau tested in compound-treated rTg4510 samples, both TCL and P3 fractions (Fig. 6a). There was an especially significant reduction in the 64-kDa (hyperphosphorylated) variant for each antibody tested; it has been reported that the 64-kDa variant was strongly correlated with neurodegeneration [53]. Importantly, transgene expression of tau was not negatively affected as there was no reduction in total tau; rather, a slight increase in the level of total tau expression was observed, which we attributed to an increased number of surviving neurons.

Next, we asked if this reduction in tau phosphorylation and deposition was a direct effect of the compound or was due to compensatory changes secondary to long-term compound treatment. To accomplish this, naïve rTg4510 mice were given a single dose of vehicle or LDN/OSU-0215111, and 24 h later, the forebrains were collected and processed for Sarkosyl isolation. We found that, even after a single dose, there was a significant decrease in pTau (AT8 and PHF1), but no change in total tau (Tau-5) or tau tangles (MC1) in TCL (Fig. 6b, open bars). Even more prominently, there was a very clear reduction of all forms of tau in the P3 fraction (Fig. 6b, black bars). This indicated a direct effect of compound mediating reduced pTau and suggested that compound may activate/inhibit a kinase/phosphatase to mediate this effect. To identify which kinase/phosphatase may be involved, forebrain TCL samples were collected from rTg4510 mice 1 h after a single dose. The phosphorylation (activation) state of most kinases that have been reported to target tau as a substrate was assessed. Of all the kinases tested, only GSK3β showed a significant change in phosphorylation state—twofold upregulation of phosphorylation at Ser9 (Fig. 6c). Furthermore, this enhanced inhibitory phosphorylation of GSK3β was maintained for up to 24 h after a single dose (Fig. 6d). This form of phosphorylation inhibits GSK3β activity [58, 59]. Therefore, inactivation of the GSK3β kinase may mediate reduced tau phosphorylation in rTg4510 mice after compound treatment.

## Discussion

In the present study, we found that LDN/OSU-0215111 exhibited a profound, disease-modifying efficacy in rTg4510 mice. At the moderate disease stage, LDN/OSU-0215111-treated mice behaved essentially indistinguishable from control mice and showed profound protection against progressive tau-mediated pathology. Even at the severe disease stage, LDN/OSU-0215111 continued to provide protection, which results in relatively normal behavior and much less severe pathology. Importantly, LDN/OSU-0215111 modulated disease directly as the delay in disease progression was sustained even after 1 month of treatment cessation. We have shown previously that this compound series has profound benefits for amyloid-β-related pathology in APP_Swe,Ind_ mice [38]. The combined results of these two efficacy studies, which represent the two most common pathologies associated with AD, strongly support LDN/OSU-0215111 as a promising clinical candidate for the treatment of AD.

The finding that the compound treatment reduced total pTau levels and insoluble forms of tau after a single dose is significant. It is known that simply turning off the MAPT*P301L transgene using DOX does not lead to a rapid reduction of aberrant tau [60]. GSK3β is differentially phosphorylated at Ser9 and Tyr216, where phosphorylation of the former inhibits the activity and of the latter maximizes the activity [61]. It is predicted that tau has approximately 85 potential phosphorylation sites and GSK3β can target many of these sites [62, 63]. The MAPT*P301L mutation in tau itself is also known to promote phosphorylation, further perpetuating a hyperphosphorylated state [64]. LDN/OSU-0215111 treatment significantly increases Ser9 phosphorylation of GSK3β, thus inhibiting the catalytic activity of GSK3β (Fig. 6c). Furthermore, this inhibition was maintained for up to 24 h after treatment (Fig. 6d) overlaying the interval between treatments resulting in persistent inhibition of GSK3β activity during this study. This may be sufficient to markedly reduce the hyperphosphorylation of tau and its subsequent deposition; however, more detailed mechanistic studies will be necessary to confirm the role that compound-mediated GSK3β inhibition plays in the phosphorylation state of mutant tau and whether compound-mediated modulation of GSK3β activity alone facilitates reduced pTau. This is a novel aspect of the mechanism of action for pyridazine-based compounds. Pyridazine derivatives are known to activate protein kinase C [42], and certain isoforms of protein kinase C are known to target GSK3β to inhibit activity; however, many other kinases are known to modulate GSK3β activity as well [65–68]. We are continuing to characterize the precise compound mechanism of action that mediates this robust reduction in pTau and tau deposition.

Previous reports have indicated that EAAT2 is decreased in rTg4510 mice [46, 69]. However, when we assessed EAAT2 expression in rTg4510 mice, we found that EAAT2 was globally upregulated in the forebrain and the increased EAAT2 was properly localized to lipid raft microdomain of the plasma membrane (Fig. 2b and Additional file 1: Figure S1c, d). This indicates that rTg4510 mice express supraphysiological levels of functional EAAT2 rather than non-functional aggregates. We have surmised this discrepancy may be related to the treatment of DOX. Application of DOX to the rodent chow silences mutant tau transgene expression [45]. In previous studies, parental mice were treated with DOX during breeding and progeny continued DOX treatment until switching to regular chow at 2 months of age [46, 69]. Decreased EAAT2 was then found at the age of 7.5 months. In our study, rTg4510 mice were never treated with DOX, therefore allowing for transgene expression throughout the lifespan. Perhaps developmental differences due to the timing of mutant tau exposure account for this discrepancy in EAAT2. When we treated adult rTg4510 mice with DOX for 3 weeks, EAAT2 protein levels were reduced in a correlated manner to tau (Additional file 1a, b), indicating that elevated EAAT2 expression may be a consequence of mutant tau expression. However, this explanation may be insufficient as EAAT2 × rTg4510 mice exhibit decreased EAAT2 but no change in total tau expression relative to rTg4510 littermates. We are further exploring this phenomenon.

Even though EAAT2 expression was not decreased in rTg4510, both transgenic overexpression of EAAT2 and LDN/OSU-0215111 treatment provided protection against many aspects of disease pathology (Fig. 1). However, LDN/OSU-0215111 provided additional benefits in rTg4510 mice that could not be accounted for by EAAT2 expression modulation alone as these benefits were not observed after EAAT2 overexpression. Behaviorally, treatment with the compound additionally normalized agitation-like hyperactivity (Figs. 3a and 4a) and, similarly, reduced gliosis (Figs. 3g, 4i) and aberrant tau expression (Figs. 3g, 6a) in rTg4510 mice while transgenic EAAT2 overexpression did not improve these phenotypes. Furthermore, for pathologies modified by both EAAT2 overexpression and compound treatment, beneficial effects observed were more robust for compound-treated rTg4510 mice than for EAAT2 × rTg4510 mice. Therefore, several of the primary benefits observed in the present therapy study may be directly attributable to the modulation of EAAT2 expression; however, it is clear that other emergent properties related to the compound mechanism of action enhance efficacy. Further research will be undertaken to better understand the multifactorial functions of the compound mechanism of action that provides these benefits.

## Conclusions

By testing pyridazine analogs in multiple models of AD, we have been able to assess the efficacy in relation to multiple different aspects of the disease with different etiologies including amyloid-β, aberrant tau, synaptic loss, and neurodegeneration which are major hallmarks of the disease. In conclusion, when intervention is given at the early symptomatic stage, LDN/OSU-0215111 significantly improves synaptic integrity, prevents progression of neurodegeneration, decreases mutant tau phosphorylation/deposition, and reduces neuroinflammation associated with tauopathy. This results in normalized cognition and behavior at moderate and severe stages in the rTg4510 mouse model of Alzheimer’s disease. Therefore, pyridazine derivatives have the potential for the treatment of AD.

## Additional file

Additional file 1: EAAT2 expression in rTg4510 mice is functional and properly localized. (a, b) At two months old, mice were treated with or without doxycycline (DOX) for three weeks to suppress mutant-tau expression. (a) Western blot analysis of forebrain total lysates showed rTg4510 mice given DOX exhibit substantial, but incomplete, reductions in the expression of all forms of tau. (b) In a similar manner, EAAT2 protein expression is reduced (partially normalized) in rTg4510 mice given DOX. (c) Hippocampal extracellular biotinylation showed that increased EAAT2 in rTg4510 mice is properly localized to the membrane. (d) The lipid-raft microdomain (fractions 3-6), which represents the functional membrane domain of EAAT2, also exhibited increased EAAT2 expression in the rTg4510 vehicle group suggesting increased functionality. Together, this suggests that increased EAAT2 in rTg4510 mice is functional and not the result of accumulation of non-functional, intracellular aggregates. (DOCX 415 kb)

## Data Availability

The datasets generated and/or analyzed during the current study are available from the corresponding author upon reasonable request.

## Abbreviations

DG: Dentate gyrus
DOX: Doxycycline
EAAT2: Excitatory amino acid transporter 2
Flot1: Flotillin-1
GFAP: Glial fibrillary acidic protein
GSK3β: Glycogen synthase kinase-3 beta
LTP: Long-term potentiation
NMDA: *N*-methyl-d-aspartate
NR2B: *N*-methyl-d-aspartate receptor 2B
NORT: Novel object recognition task
PAP: Perisynaptic astrocytic processes
PSD-95: Postsynaptic density 95
pTau: Phosphorylated tau
P3: Tris-buffered saline extractable, Sarkosyl-insoluble fraction
TCL: Total cell lysate
