# Supplementary figures and images for: Enhancement of tripartite synapses as a potential therapeutic strategy for Alzheimer’s disease: a preclinical study in rTg4510 mice
Source: Alzheimers Res Ther. 2019 Aug 23;11:75. doi: 10.1186/s13195-019-0530-z (PMC6706914; doi:10.1186/s13195-019-0530-z)

Additional files

**
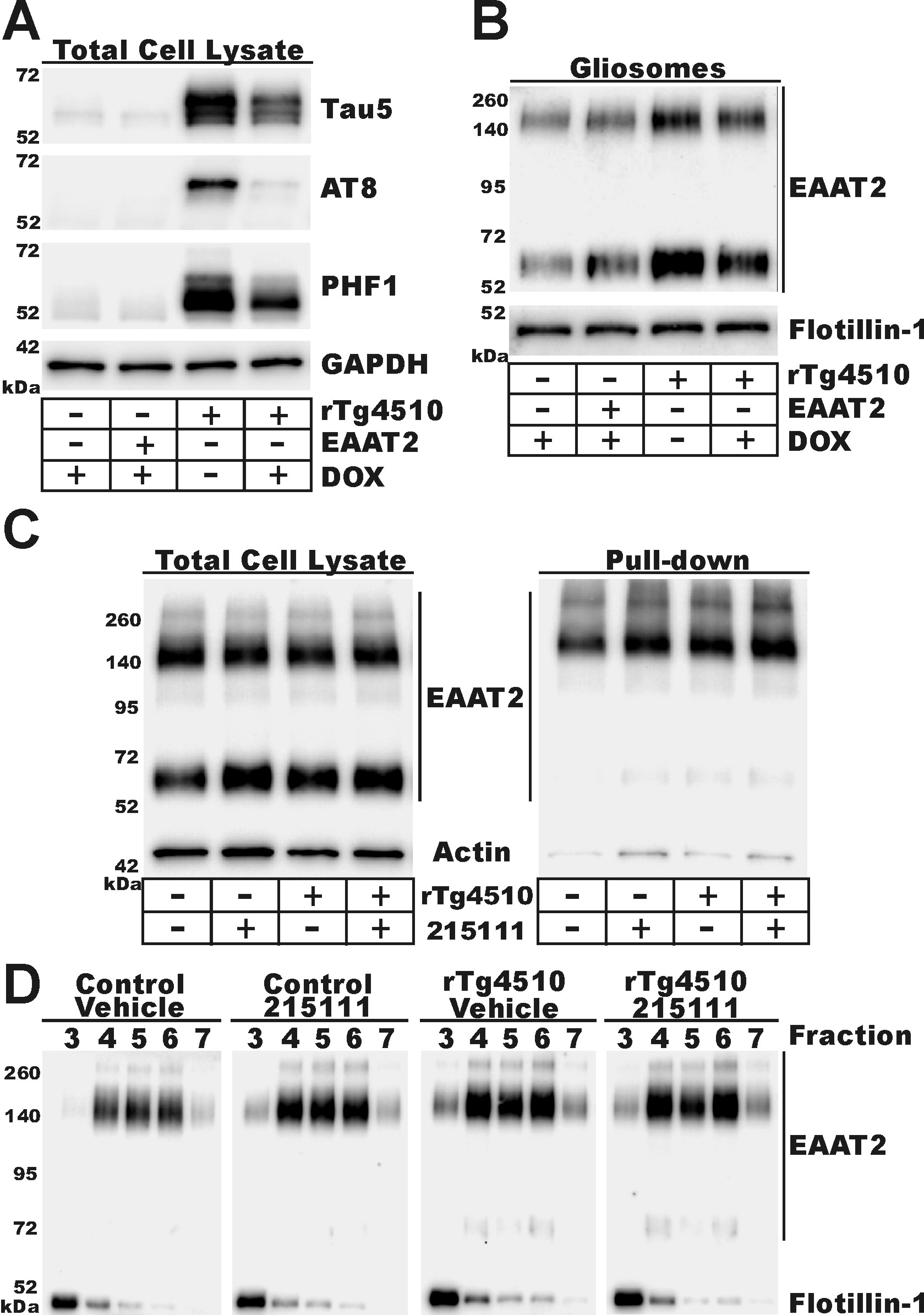
**

Supplement: Supplementary file 1 — EAAT2 expression in rTg4510 mice is functional and properly localized. (a, b) At two months old, mice were treated with or without doxycycline (DOX) for three weeks to suppress mutant-tau expression. (a) Western blot analysis of forebrain total lysates showed rTg4510 mice given DOX exhibit substantial, but incomplete, reductions in the expression of all forms of tau. (b) In a similar manner, EAAT2 protein expression is reduced (partially normalized) in rTg4510 mice given DOX. (c) Hippocampal extracellular biotinylation showed that increased EAAT2 in rTg4510 mice is properly localized to the membrane. (d) The lipid-raft microdomain (fractions 3-6), which represents the functional membrane domain of EAAT2, also exhibited increased EAAT2 expression in the rTg4510 vehicle group suggesting increased functionality. Together, this suggests that increased EAAT2 in rTg4510 mice is functional and not the result of accumulation of non-functional, intracellular aggregates. (DOCX 415 kb) [file 13195_2019_530_MOESM1_ESM.docx]
